# S. W. Noble, M.D., Bloomington, Ill.

**Published:** 1871-05

**Authors:** 


At a called meeting of the McLean County Medical Society, on Wednesday, March 15, 1871, at the office of Drs. Bradley & Laughlin, in Bloomington, Illinois, to take action with regard to the death of Dr. S. W. Noble, the following preamble and resolutions were unanimously adopted:

*Whereas*—We learn with sadness that our much loved companion—Dr. S. W. Noble—after a protracted and painful illness, which he endured with great fortitude and patience, died on yesterday, at 7 o'clock P.M.

*Resolved*—That in his untimely death our Society has lost one of its most honored and useful members; the community a man of superior character and influence; and his family a devoted husband and affectionate father.

*Resolved*—That we will ever cherish the memory of his ardent attachment to the interests of our Society, and his unremitting devotion to the cause of our noble profession, to whose dignity and practice he consecrated all his powers and all his great talents, and in the service of which he spent his life.

*Resolved*—That we commend Dr. Noble's example, in that his devotion to his beloved science prompted him to express his desire that a *post mortem* examination of his body be made, in order to confirm or correct the diagnosis of his case.

*Resolved*—That we hereby tender to his bereaved family and friends our sincere sympathy in this their sad and irreparable loss; and that a copy of these resolutions be published in the city papers, and in the Chicago journals, and also in the *Western Lancet and Observer*, and that a copy be handed to the afflicted wife of our deceased brother.

A. H. LUCE,

R. G. LAUGHLIN,

T. E. WORRELL,

} *Committee.*

R. D. BRADLEY,

*Secretary.*

